# A TrkB–STAT3–miR-204-5p regulatory circuitry controls proliferation and invasion of endometrial carcinoma cells

**DOI:** 10.1186/1476-4598-12-155

**Published:** 2013-12-09

**Authors:** Wei Bao, Hui-Hui Wang, Fu-Ju Tian, Xiao-Ying He, Mei-Ting Qiu, Jing-Yun Wang, Hui-Juan Zhang, Li-Hua Wang, Xiao-Ping Wan

**Affiliations:** 1 Department of Obstetrics and Gynecology, International Peace Maternity and Child Health Hospital, Shanghai Jiao Tong University School of Medicine, No. 910, Hengshan Road, Shanghai 200030, China; 2 Department of Obstetrics and Gynecology, Shanghai First People’s Hospital, Shanghai Jiao Tong University School of Medicine, No. 100, Haining Road, Shanghai 200080, China; 3 Department of the Centre of Research Laboratory, International Peace Maternity and Child Health Hospital, Shanghai Jiao Tong University School of Medicine Shanghai, China; 4 Department of Pathology, International Peace Maternity and Child Health Hospital, Shanghai Jiao Tong University School of Medicine Shanghai, China

**Keywords:** Endometrial carcinoma, miR-204-5p, TrkB, STAT3, Regulatory circuitry, Proliferation, Invasion, Prognosis

## Abstract

**Background:**

We previously identified TrkB as an oncogene involved in promoting metastasis in endometrial carcinoma (EC). Here, we sought to delineate the effect of changes in TrkB expression on the global profile of microRNAs (miRNAs) in EC cells and further investigated the correlation between the expression of certain miRNA and TrkB in the clinicopathologic characteristics of EC patients.

**Methods and results:**

Using quantitative reverse transcription-PCR (qRT-PCR), we found that expression of *TrkB* mRNA has no significant difference in transcript levels between normal endometrium and EC cells captured by laser capture microdissection, while immunohistochemistry results demonstrated a markedly higher expression of TrkB protein in EC tissues. The microRNA array showed that ectopic overexpression and knockdown of TrkB expression caused global changes in miRNA expression in EC cells. qRT-PCR results showed that elevated TrkB repressed miR-204-5p expression in EC cells. Furthermore, immunoblotting assays revealed that TrkB overexpression in Ishikawa^TrkB^ cells noticeably increased JAK2 and STAT3 phosphorylation, which, however, was aborted by *TrkB* knockdown in HEC-1B^shTrkB^ cells. Moreover, ChIP assays showed that phospho-STAT3 could directly bind to STAT3-binding sites near the *TRPM3* promoter region upstream of *miR-204-5p*. Interestingly, using bioinformatics analysis and luciferase assays, we identified TrkB was a novel target of miR-204-5p. Functionally, the MTT assays, clonogenic and Transwell assays showed that miR-204-5p significantly suppressed the clonogenic growth, migration and invasion of EC cells. Furthermore, miR-204-5p also inhibited the growth of tumor xenografts bearing human EC cells. Importantly, we found lower miR-204-5p expression was associated with advanced FIGO stages, lymph node metastasis and probably a lower chance for survival in EC patients.

**Conclusions:**

This study uncovers a new regulatory loop involving TrkB/miR-204-5p that is critical to the tumorigenesis of EC and proposes that reestablishment of miR-204-5p expression could be explored as a potential new therapeutic target for this disease.

## Background

Endometrial carcinoma is the most common gynecological malignancy worldwide. For 2013, in the USA alone, 49,560 persons will be newly diagnosed with endometrial carcinoma and 8,190 persons will die of this disease [1]. Hysterectomy with or without adjuvant treatment has been recommended for local endometrial carcinoma, yielding a 5-year survival rate of approximately 96%. However, 30% endometrial carcinoma cases are not diagnosed until regional or distant metastasis is present, resulting in a much more dismal outcome [2]. Thus, identification of additional molecular and cellular mechanisms responsible for tumorigenesis and progression of endometrial cancer, and discovery of novel diagnostic and prognostic biomarkers and development of novel therapeutic strategies for endometrial cancer are critical to improving the diagnosis and prognosis of endometrial cancer patients.

Endometrial cancer was originally classified according to a dualistic model [3]. More recently, this model has been challenged because tumors seen in daily practice occasionally show overlapping or combined morphologic and molecular characteristics of both classification types or exhibit ambiguous features [4]. In endometrial cancer, myometrial invasion and lymph node metastasis are considered the most important prognostic factors [5]. For these processes to occur, epithelial tumor cells need to undergo an epithelial to mesenchymal transition (EMT) [6]. Neurotrophic receptor tyrosine kinase B (TrkB) has been shown to be a key regulator of oncogenesis and tumor progression in various human cancer types including cancer of the lungs and breast [7,8]. We have also previously demonstrated a novel role of TrkB in promoting EMT and resistance to anoikis [9]. As an additional receptor tyrosine kinase, TrkB activates diverse downstream signaling cascades that ultimately induce cellular proliferation and pro-survival mechanisms through the AKT, STAT3 and MAPK signaling pathways [10,11].

MicroRNAs (miRNAs) are small non-coding ribonucleic acids (RNAs) of approximately 22 bp that induce RNA interference by base-pairing with the 3′ untranslated region (UTR) of a complementary messenger RNA (mRNA), which triggers either mRNA translational repression or RNA degradation [12]. Approximately 20–30% of all genes are targeted by miRNAs, and a single miRNA may target as many as 200 genes [13]. In human cancers, specific miRNAs are expressed in different tissues, and changes in the regulation of gene expression have been associated with carcinogenesis [14], including endometrial cancer [15]. Furthermore, miRNAs cooperatively function with certain transcription factors in the regulation of mutual sets of target genes, allowing coordinated modulation of gene expression both transcriptionally and posttranscriptionally. Specifically, a recurring network motif has been revealed in which a transcription factor regulates a miRNA with which it cooperates in regulating a common set of targets [16]. These observations prompted us to hypothesize that specific miRNAs may control TrkB expression posttranscriptionally in endometrial cancer progression.

Here, we report the identification of a set of miRNAs repressed by TrkB in two endometrial cancer cell lines by comprehensive miRNA profiling. One candidate miRNA of interest, *miR-204-5p*, is located at the cancer-associated genomic region *9q21.1–q22.3* locus and is known to be significantly dysregulated in broad tumor types, including breast, prostate, and kidney cancers [17], suggesting a role for *miR-204-5p* as a tumor suppressor gene. We demonstrate a role for *miR-204-5p* in endometrial cancer and also shed light on a novel posttranscriptional regulatory circuit in which TrkB induces the activation of STAT3 to regulate the expression of miR-204-5p, which in turn, directly modulates TrkB expression in endometrial cancer cells. These results establish miR-204-5p as a novel TrkB regulator and a potential therapeutic target for EC.

## Results

### TrkB overexpression is associated with global changes in miRNA expression in endometrial cancer cells

**Figure 1:**
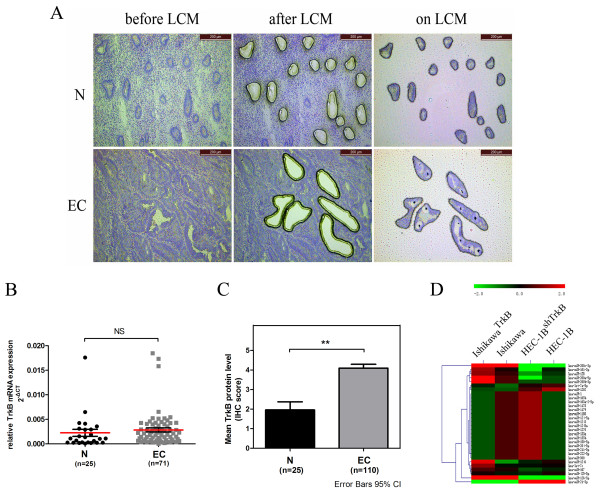
**TrkB overexpression is associated with global changes in miRNA expression in endometrial cancer cells. (A)** Laser capture microdissection (LCM) of normal (N) endometrial cells and endometrial cancer (EC) cells. 100×. **(B)** Quantitative reverse transcription polymerase chain reaction (qRT-PCR) of TrkB in endometrial cancer specimens (n = 71) and normal tissues (n = 25). TrkB expression was normalized against β-actin. Data are presented as individual samples with the line indicating the mean +/− SD. NS, *P* > 0.05. **(C)** Immunostaining scores of TrkB in normal endometrium (n = 25) and endometrial cancer specimens (n = 110). Data are presented as mean +/− SD. **, *P* < 0.01. **(D)** MicroRNA array analysis of miRNA expression in endometrial cancer cells. Hierarchical clustering of the expression values for 1347 mature miRNAs of Ishikawa^TrkB^ versus Ishikawa cells and HEC-1B^shTrkB^ versus HEC-1B cells.

We examined TrkB protein and mRNA expression in the normal endometrium and endometrial cancer tissues using laser capture microdissection (LCM)/quantitative reverse transcription polymerase chain reaction (qRT-PCR) and immunohistochemistry. Our RT-PCR assays of normal and endometrial cancer cells captured by LCM (Figure 1A) revealed that mRNA transcript levels of *TrkB* appeared to be higher in the tumors than in the normal specimens, but overall the difference in *TrkB* mRNA expression between endometrial cancer tissues and the normal endometrium was not statistically significant (*P* > 0.05) (Figure 1B). Immunohistochemistry, on the other hand, demonstrated a markedly higher expression of TrkB in endometrial cancer tissues compared with the normal endometrium (*P* < 0.0001) (Figure 1C). The data suggest that TrkB is upregulated mainly at the posttranscriptional level in human endometrial cancer tissues.

We were interested in whether changes in TrkB expression impacted on the global profile of miRNA expression in endometrial cancer cells. Our microRNA array consisting of 1347 capture probes for mature human miRNAs showed marked changes in the expression of 98 miRNAs in HEC-1B^shTrkB^ cells whose TrkB expression was suppressed by short hairpin RNA (shRNA) against *TrkB* compared to HEC-1B cells (Figure 1D). TrkB overexpression also caused marked changes in 126 miRNAs in Ishikawa^TrkB^ cells ectopically expressing TrkB compared to Ishikawa cells (Figure 1D). Consistently, 76 miRNAs were found among the differentially expressed miRNAs in both HEC-1B^shTrkB^ cells (77.6%, 76/98) and Ishikawa^TrkB^ cells (60.3%, 76/126) (Table 1).

**Table 1:** Microarray profiling of changes in global miRNA expression in endometrial cancer cells with high or low TrkB expression

**MiRNA**	**Fold change**	**MiRNA**	**Fold change**
**Ishikawa**^**TrkB **^**vs. Ishikawa**		**HEC-1B**^**shTrkB **^**vs. HEC-1B**	
*Downregulated*		*Upregulated*	
hsa-miR-1	−1.53	hsa-miR-1	1.84
hsa-miR-103b	−1.53	hsa-miR-103b	1.84
hsa-miR-103a-2-5p	−1.53	hsa-miR-103a-2-5p	1.84
hsa-miR-1178	−1.53	hsa-miR-1178	1.84
hsa-miR-1179	−1.53	hsa-miR-1179	1.84
hsa-miR-1180	−1.53	hsa-miR-1180	1.84
hsa-miR-124-5p	−1.53	hsa-miR-124-5p	1.84
hsa-miR-1243	−1.53	hsa-miR-1243	1.84
hsa-miR-1245a	−1.53	hsa-miR-1245a	1.84
hsa-miR-1270	−1.53	hsa-miR-1270	1.84
hsa-miR-133a	−1.53	hsa-miR-133a	1.84
hsa-miR-133b	−1.53	hsa-miR-133b	1.84
hsa-miR-150-5p	−1.53	hsa-miR-150-5p	1.84
hsa-miR-200c-3p	−1.56	hsa-miR-200c-3p	1.84
hsa-miR-204-5p*	−1.53	hsa-miR-204-5p*	1.84
hsa-miR-21-3p	−5.95	hsa-miR-21-3p	1.97
hsa-miR-211-5p*	−1.53	hsa-miR-211-5p*	1.84
hsa-miR-222-5p	−1.53	hsa-miR-222-5p	1.84
hsa-miR-300	−1.53	hsa-miR-300	1.84
*Upregulated*		*Downregulated*	
hsa-let-7a-5p	1.52	hsa-let-7a-5p	−1.51
hsa-let-7c	1.53	hsa-let-7c	−1.66
hsa-miR-101-3p	1.52	hsa-miR-101-3p	−2.13
hsa-miR-107	1.52	hsa-miR-107	−1.53
hsa-miR-1202	1.53	hsa-miR-1202	−1.52
hsa-miR-1246	2.83	hsa-miR-1246	−1.68
hsa-miR-126-3p	5.39	hsa-miR-126-3p	−2.66
hsa-miR-128	1.69	hsa-miR-128	−1.51
hsa-miR-200a-5p	2.05	hsa-miR-200a-5p	−2.04
hsa-miR-200b-3p	1.87	hsa-miR-200b-3p	−1.51
hsa-miR-425-5p	1.51	hsa-miR-425-5p	−1.59

*predicted to bind to *TrkB* 3′-UTR by TargetScan, Pictar and MiRanda.

### *MiR-204-5p* is a negative modulator of TrkB expression in endometrial cancer cells

**Figure 2:**
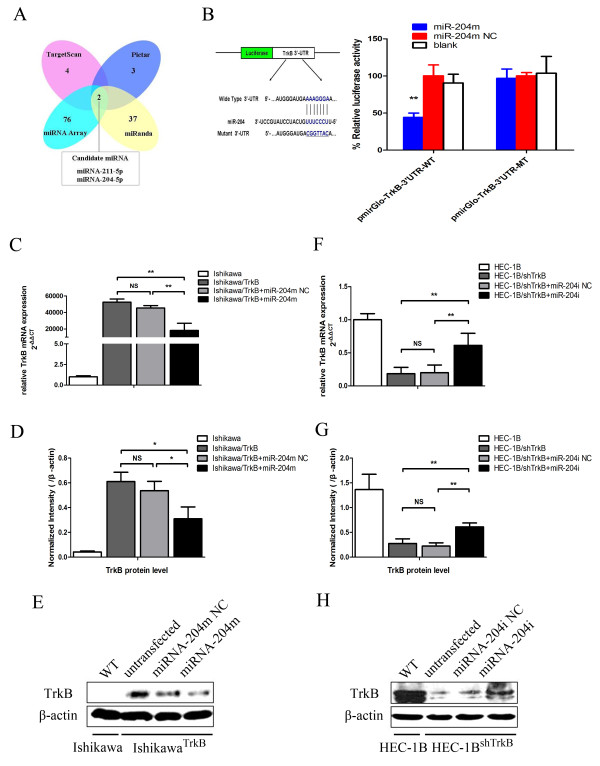
***MiR-204-5p *is a negative modulator of TrkB expression in endometrial cancer cells. (A)** Schema of selection of candidate miRNAs (*miR-211-5p* and *miR-204-5p*) targeting *TrkB* using three online target-prediction programs (TargetScan, Pictar and miRanda) and the microarray data. **(B)** Left panel: Schema of the construction of pMIRGLO-TrkB-3′UTR-WT and pMIRGLO-TrkB-3′UTR-MT vectors. The mutant binding site is underlined. Right panel: 293 T cells were co-transfected with pMIRGLO-TrkB-3′UTR-WT or pMIRGLO-TrkB-3′UTR-MT and miR-204-5p mimic (miR-204 m) or scrambled miRNA control (miR204m NC). Luciferase activities are shown as mean ± SD of at least three independent experiments done in triplicate. **P < 0.01. **(C)** Ishikawa^TrkB^ cells were transfected with miR-204 m NC or miR-204 m. TrkB expression was examined by RT-PCR and normalized against β-actin. Data are shown as mean ± SD of at least three independent experiments.** *P* < 0.01; NS *P* > 0.05. **(D)** Cells were transfected as in **(C)** and TrkB expression was determined by Western blotting assays, which were quantified by densitometry of triplicate experiments (* *P* < 0.05; NS *P* > 0.05). β-actin was included as an internal control. Representative blots are shown in **(E). (F)** HEC-1B^shTrkB^ cells were transfected with a miR-204 inhibitor (miR-204i) or a scrambled control inhibitor (miR-204i NC) or with miR-204 inhibitor (miR-204i). TrkB expression was examined by RT-PCR and normalized against β-actin. Data are shown as mean ± SD of at least three independent experiments.** *P* < 0.01; NS *P* > 0.05. **(G)** Cells were transfected as in **(F)** and TrkB expression was determined by Western blotting assays, which were quantified by densitometry of triplicate experiments (** *P* < 0.01; NS *P* > 0.05). β-actin was included as an internal control. Representative blots are shown in **(H)**.

Separately, we surveyed the 3′-UTR of the *TrkB* promoter using three target-prediction algorithms, TargetScan, Pictar and MiRanda, to identify candidate miRNAs which may act as posttranscriptional modulators of TrkB expression. TargetScan, Pictar and MiRanda revealed 4, 3 and 37 candidate miRNAs, respectively (Figure 2A). Examination of the 76 differentially expressed miRNAs as identified by microarray analysis showed that miR-211-5p and miR-204-5p were the only two candidate miRNAs that were also identified by all three target-prediction algorithms to potentially bind to the 3′-UTR of the *TrkB* (Figure 2A). Furthermore, the miR-204-5p targeting site within the 3′UTR of *TrkB* (position 457–464) was highly conserved across five mammalian species (Additional file 1: Figure S1A). These intriguing findings suggest that miR-211-5p and miR-204-5p and TrkB are likely mutual modulators of their expression.

MiR-204-5p is reportedly dysregulated in endometrial carcinoma [18,19]. To examine whether miR-204-5p modulated TrkB expression, we constructed vectors containing a wildtype or mutant TrkB 3′UTR directly fused downstream of the Firefly luciferase reporter gene (Figure 2B). The wildtype or mutant vector was co-transfected into 293 T cells with miR-204-5p mimic (miR-204 m) or its scrambled control (miR-204 m NC). Co-transfection assays showed that, compared with the scrambled miRNA control, miR-204 m significantly decreased the relative luciferase activities of 293 T cells transfected with the wildtype *TrkB* 3′UTR (*P* < 0.01) (Figure 2B). No reduction in luciferase activities was observed of 293 T cells transfected with the mutant *TrkB* 3′UTR.

Our finding that TrkB may be targeted by miR-204-5p led us to further delineate the actions of miR-204-5p on TrkB in endometrial cancer cells. Our RT-PCR assays revealed that Ishikawa^TrkB^ cells transfected with miR-204-5 m had markedly reduced *TrkB* mRNA transcript levels compared to Ishikawa^TrkB^ cells transfected with scrambled miRNA (*P* < 0.01) (Figure 2C). Immunoblotting assays also showed that Ishikawa^TrkB^ cells transfected with miR-204 m had markedly reduced TrkB levels (*P* < 0.05) (Figure 2D and E). Furthermore, HEC-1B^shTrkB^ cells transfected with a miR-204-5p inhibitor (miR-204i) had markedly higher *TrkB* mRNA transcript levels than HEC-1B^shTrkB^ cells transfected with scrambled miRNA (*P* < 0.01) (Figure 2F). Our immunoblotting assays additionally showed that HEC-1B^shTrkB^ cells transfected with miR-204i also had noticeably increased levels of TrkB (*P* < 0.01) (Figure 2G and H). These findings indicated that *miR-204-5p* negatively regulated the expression of TrkB.

### TrkB represses *miR-204-5p* expression by activating the JAK2/STAT3 pathway *in vitro*

**Figure 3:**
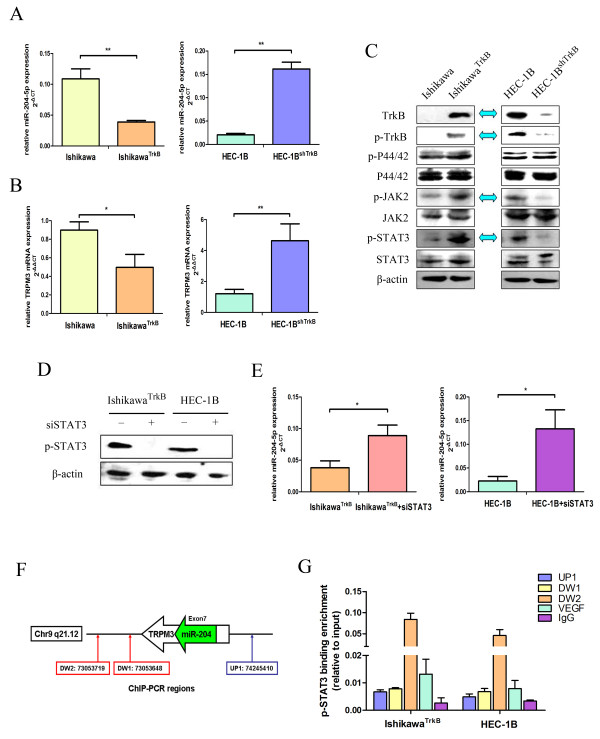
**TrkB represses *miR-204-5p *expression by activating the JAK2/STAT3 pathway. (A)** TrkB inversely regulates *miR-204-5p* expression and **(B)***TRPM3* mRNA expression in endometrial cancer cells. *MiR-204-5p* and *TRPM3* mRNA levels in Ishikawa and Ishikawa^TrkB^ cells and HEC-1B and HEC-1B^shTrkB^ cells were measured by quantitative RT-PCR. *MiR-204-5p* expression was normalized against U6B and *TRPM3* mRNA expression was normalized against β-actin. Data are expressed as mean ± SD of at least three independent experiments. **P < 0.01 and *P < 0.05. **(C)** TrkB, phospho-TrkB, phospho-P44/42, P44/42, JAK2, STAT3, phospho-JAK2 and phospho-STAT3 in Ishikawa and Ishikawa^TrkB^ cells and HEC-1B and HEC-1B^shTrkB^ cells were assessed by Western blotting assays. Representative blots are shown. Blue arrows show the opposite changes in the two cell lines. **(D)** Ishikawa^TrkB^ and HEC-1B cells were transfected with siSTAT3, and phospho-STAT3 was assessed by Western blotting assays. **(E)** Inhibition of STAT3 increases *miR-204-5p* expression in Ishikawa^TrkB^ and HEC-1B cells. Data are expressed as mean ± SD of at least three independent experiments. *P < 0.05. **(F)** Schematic representation of the *miR-204-5p* locus and its host gene, *TRPM3*. STAT3-binding sites around the *TRPM3* start site (UP1, DW1, and DW2) are predicted by *in silico* analysis. Chromatin immunoprecipitation assays were performed using anti-phospho-STAT3 antibody or rabbit isotype IgG. Immunoprecipitated DNA fragments were examined by quantitative real-time PCR **(G)**. Data in **(G)** represent mean ± SD of at least three independent experiments performed in triplicate. *VEGF* was used as positive control and *IgG* as negative control.

Our microRNA microarray showed that miR-204-5p was downregulated in Ishikawa^TrkB^ cells. Further examination by qRT-PCR assays revealed that miR-204-5p expression was noticeably suppressed in Ishikawa^TrkB^ cells (vs. Ishikawa cells, *P* < 0.01), but markedly enhanced in HEC-1B^shTrkB^ cells (vs. HEC-1B cells, *P* < 0.01) (Figure 3A). These results further corroborated the microRNA array findings and indicated that TrkB suppresses miR-204-5p expression. Our qRT-PCR assays also showed that the mRNA transcript levels of *TRPM3*, which shares the same regulatory motif for transcription with miR-204 [17], were significantly decreased in Ishikawa^TrkB^ cells (*P* < 0.05), but markedly elevated in HEC-1B^shTrkB^ cells (*P* < 0.01) (Figure 3B), lending support to an inhibitory role of TrkB at the *TRPM3/miR-204-5p* locus.

The TrkB signaling pathway overlaps with the ERK/MAPK or JAK2/STAT3 signaling pathway in myeloma or endometrial cancer cells [20,21], which prompted us to examine whether TrkB suppressed miR-204-5p expression via these signaling pathways. Our immunoblotting assays revealed that TrkB overexpression in Ishikawa^TrkB^ cells noticeably increased the phosphorylation of JAK2 and STAT3, which, however, was aborted by *TrkB* knockdown in HEC-1B^shTrkB^ cells (Figure 3C). No obvious activation was found by TrkB overexpression in ERK/MAPK pathway (Figure 3C), and effect of its inhibition (U0126 10 μM) on miR-204-5p expression (Additional file 2: Figure S2A-C). Moreover, inhibition of STAT3 by small interference RNA (siRNA) (Figure 3D) elevated the miR-204-5p expression in Ishikawa^TrkB^ and HEC-1B cells (*P* < 0.05) (Figure 3E), indicating that, indeed, TrkB activates the JAK2/STAT3 pathway in endometrial cancer cells. We further examined whether phospho-STAT3 could bind to three putative STAT-binding sites that are located near the *TRPM3* promoter region upstream of *miR-204-5p*[22] (Figure 3F). Our ChIP assays showed that phospho-STAT3 could bind to all the three putative STAT-binding sites in Ishikawa^TrkB^ and HEC-1B cells, with the greatest binding to DW2 (Figure 3G). These data together suggest that TrkB overexpression upregulates the phosphorylation levels of STAT3, which binds to the STAT-binding sites near the *TRPM3* promoter region upstream of *miR-204-5p*, leading to repression of miR-204 in endometrial cancer cells.

### TrkB promotes while *miR-204-5p* suppresses the clonogenic growth, migration and invasion of endometrial cancer cells *in vitro*

**Figure 4:**
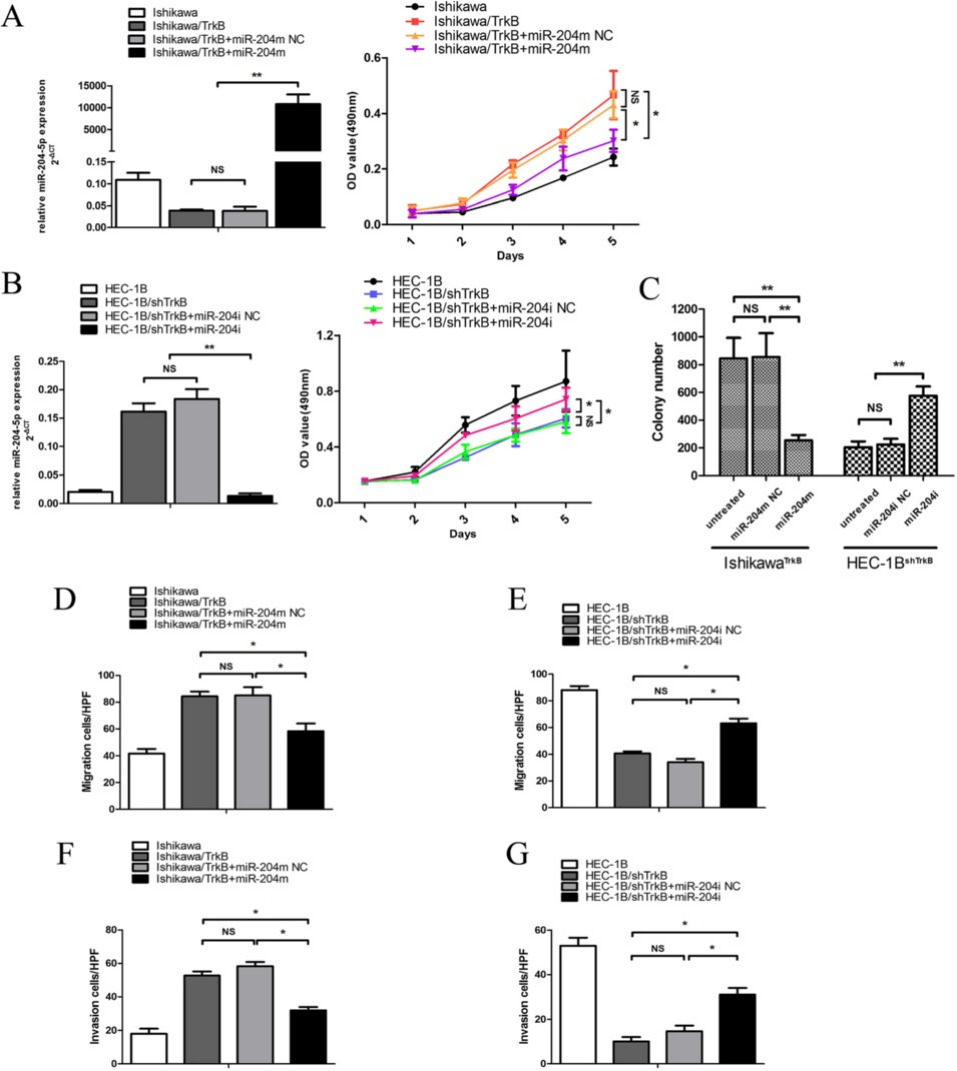
**TrkB promotes while miR-204-5p suppresses the clonogenic growth, migration and invasion of endometrial cancer cells. (A)** Left panel: Ishikawa^TrkB^ cells were transfected with miR-204 m or miR-204 m NC. MiR-204-5p expression was measured by *Taq*Man PCR assays 72 h post transfection and normalized against U6B. Data are shown as mean ± SD of at least three independent experiments. ** *P* < 0.01; NS, *P* > 0.05. Right panel: The growth curves of Ishikawa cells were plotted by MTT assays. Data are shown as mean ± SD of at least three independent experiments. * *P* < 0.05; NS, *P* > 0.05. **(B)** Left panel: HEC-1B^shTrkB^ cells were transfected with miR-204i or miR-204i NC. MiR-204-5p expression was measured by *Taq*Man PCR assays 72 h post transfection and normalized against U6B. Data are shown as mean ± SD of at least three independent experiments. ** *P* < 0.01 vs. miR-204i; NS, *P* > 0.05. Right panel: The growth curves of HEC-1B cells were plotted by MTT assays. * *P* < 0.05; NS, *P* > 0.05. **(C)** Ishikawa^TrkB^ cells were transfected as in **(A)** and HEC-1B^shTrkB^ cells as in **(B)**. Colony formation was observed. Data are shown as mean ± SD of at least three independent experiments. ** *P* < 0.01; NS *P* > 0.05. **(D** to **G)** Ishikawa^TrkB^ cells were transfected with miR-204 m or miR-204 m NC and HEC-1B^shTrkB^ cells with miR-204i or miR-204i NC. Transwell and cell invasion assays were done as described in Methods. The number of migrated cells is shown in (**D** and **E**, * *P* < 0.05; NS, *P* > 0.05). The number of invasive cells is shown in (**F** and **G**, * *P* < 0.05; NS, *P* > 0.05).

To investigate whether miR-204-5p modulated the growth of endometrial cancer cells, we transfected Ishikawa^TrkB^ cells with miR-204 m or its scrambled control and HEC-1B^shTrkB^ cells with miR-204i or its scrambled control. The MTT assays showed that compared with Ishikawa cells, Ishikawa^TrkB^ cells, which had markedly reduced miR-204-5p as measured by *Taq*Man PCR assays (Figure 4A, left panel), exhibited significantly increased growth (*P* < 0.05) (Figure 4A, right panel). Treatment with miR-204 m, however, significantly attenuated the growth of Ishikawa^TrkB^ cells (vs. scrambled control or non-transfected cells, *P* < 0.05) (Figure 4A, right panel). Conversely, compared to HEC-1B cells, HEC-1B^shTrkB^ cells, which had markedly increased miR-204-5p as measured by *Taq*Man PCR assays (Figure 4B, left panel), showed markedly reduced growth (*P* < 0.05) (Figure 4B, right panel). Treatment with miR-204i partially and yet significantly rescued the growth of HEC-1B^shTrkB^ cells (vs. scrambled control or non-transfected cells, *P* < 0.05) (Figure 4B, right panel). Furthermore, the clonogenic assays showed that miR-204 m caused a greater than 50% reduction in the number of colonies compared with that of Ishikawa^TrkB^ cells or Ishikawa^TrkB^ cells transfected with the scrambled control (*P* < 0.01 in both) (Figure 4C, Additional file 3: Figure S3A). By contrast, miR-204i noticeably increased the number of colonies compared with that of HEC-1B^shTrkB^ cells or HEC-1B^shTrkB^ cells transfected with the scrambled control (*P* < 0.01 in both) (Figure 4C, Additional file 3: Figure S3A). These results suggest that TrkB promotes while miR-204-5p suppresses the clonogenic growth of endometrial cancer cells.

We further assessed whether miR-204-5p modulated the migratory and invasive capacity of endometrial cancer cells. Our Transwell assays showed that Ishikawa^TrkB^ cells displayed an enhanced capacity of migration compared to Ishikawa cells, which, however, was markedly abated by miR-204 m (vs. non-transfected or scrambled control, *P* < 0.05) (Figure 4D, Additional file 3: Figure S3B). Conversely, HEC-1B^shTrkB^ cells exhibited reduced migration capacity compared to HEC-1B cells, which was enhanced by miR-204i (vs. non-transfected or scrambled control, *P* < 0.05) (Figure 4E, Additional file 3: Figure S3B). Furthermore, similar findings were observed in invasion assays of Ishikawa^TrkB^ cells and HEC-1B^shTrkB^ cells (Figure 4F and G, Additional file 3: Figure S3C). These results together demonstrate that TrkB increases while miR-204-5p suppresses the clonogenic growth, migration and invasion of endometrial cancer cells *in vitro.*

### TrkB is a functionally important target of *miR-204-5p* involved in the clonogenic growth, migration and invasion of endometrial cancer cells in vitro

**Figure 5:**
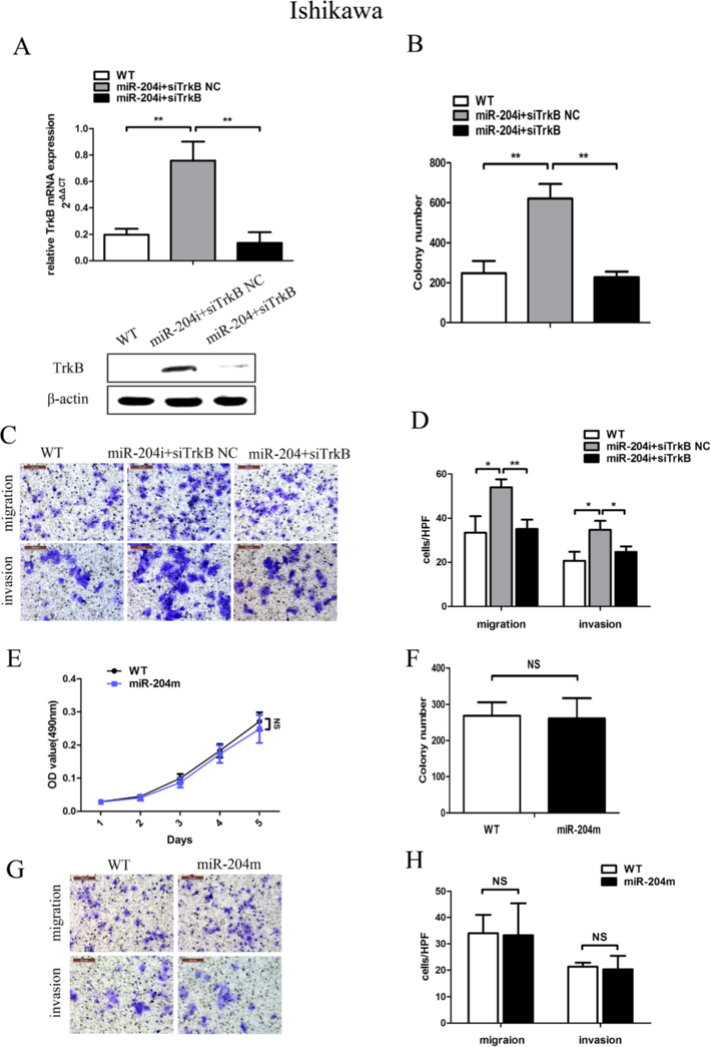
**TrkB is a functionally target of *miR-204-5p *involved in the clonogenic growth, migration and invasion of endometrial cancer cells. (A)** Upper panel, Ishikawa cells were co-transfected with miR-204i or its scrambled control and siTrkB or its scrambled control. Expression of TrkB mRNA in Ishikawa cells was measured by RT-PCR assays at 48 h posttransfection. Lower panel, TrkB protein levels were measured by western blotting assays at 72 h posttransfection. Cologenic assays were done as described in Methods and are shown in **(B)**. **(C)** Migrated and invasive cells on the lower surface of the Transwell filter were stained and photographed, 200×. The number of migrated and invasive cells is shown in **(D)**. **(E)** Ishikawa cells were transfected with miR-204 m, and the growth curves of Ishikawa cells were plotted by MTT assays. **(F)** Colony formation was observed. Transwell and cell invasion assays were done as described in Methods. The number of migrated and invasive cells is shown in **(G** and **H)**. In all the panels, data are shown as mean ± SD of at least three independent experiments. * *P* < 0.05, ** *P* < 0.01; NS *P* > 0.05.

MiRNAs can target a series of mRNAs representing anywhere from several to hundreds of genes. To address whether the phenotypic effects of miR-204-5p expression are predominately due to the suppression of TrkB, rather than one of its other cellular targets, we additionally examined whether miR-204-5p and TrkB functioned in the same pathway in modulating clonogenic growth, migration and invasion of endometrial cancer cells. We abolished miR-204-5p activity by transfecting Ishikawa cells with miR-204i. The RT-PCR and Western blotting assays showed that in miR-204i-transfected cells, co-transfection with siTrkB significantly reduced TrkB expression as compared to co-transfection with siTrkB NC (Figure 5A). Furthermore, in the absence of miR-204-5p activity, the number of colonies in Ishikawa cells was increased (*P* < 0.01), while silencing of *TrkB* by specific siRNA resulted in a marked reduction in the number of colonies compared with the scrambled control (*P* < 0.01) (Figure 5B); and a similar reduction was observed in the number of migrated and invasive cells (*P* < 0.01) (Figure 5C and D). Furthermore, we chose Ishikawa cells, who do not express TrkB to see if miR-204-5p alone exerted any functional effect on EC cell lines. As expected, cells treated with miR-204 m had no obvious change in cellular proliferation, colony formation, migration and invasion compared with wildtype Ishikawa cells (Figure 5E-H). Together, these results suggest that TrkB is a functionally important downstream target of *miR-204-5p* that is involved in the clonogenic growth, migration and invasion of endometrial carcinoma cells.

### *MiR-204-5p* inhibits the growth of mouse xenografts bearing human endometrial carcinoma cells

**Figure 6:**
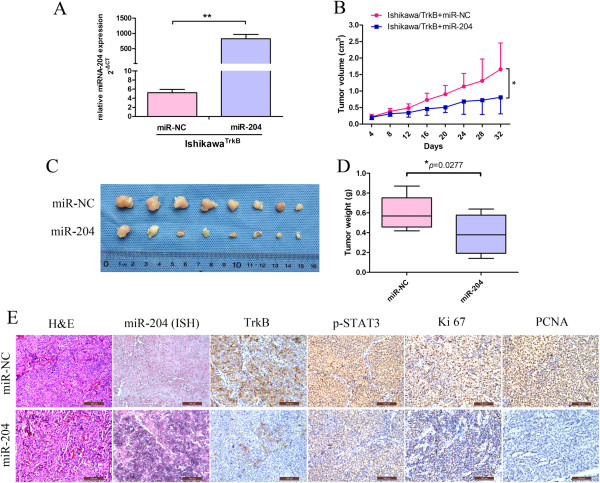
***MiR-204-5p *inhibits the growth of mouse xenografts bearing human endometrial carcinoma cells. (A)** Ishikawa^TrkB^ cells were transfected with lentiviruses encoding miR-204 or its scrambled control (NC). MiR-204 was detected by *Taq*Man PCR and normalized against U6B. Data are expressed as mean ± SD of at least three independent experiments. ** *P* < 0.01. **(B)** Tumor growth curve in nude mice bearing Ishikawa^TrkB^ cells transfected with lentiviruses encoding miR-204 or its scrambled control. Data are expressed as mean ± SD of 8 mice. * *P* < 0.05. **(C)** Photograph of tumor xenografts. **(D)** Tumor weights are expressed as mean ± SD of tumors from panel C. *P < 0.05. **(E)** Representative H&E staining histopathology of miR-204 NC and miR-204 tumor tissues in mice (left panels). MiR-204 expression was detected by *in situ* hybridization (second panels), and TrkB, p-STAT3, Ki 67 and PCNA expression was detected by immunohistochemistry (right panels) (magnification, 200×). Results are representative of three independent experiments.

We were interested in whether miR-204-5p suppressed the growth of xenografts bearing human endometrial carcinoma cells. We transfected Ishikawa^TrkB^ cells with miR-204-5p precursor or its scrambled control using lentiviruses (Figure 6A, Additional file 4: Figure S4A). Compared to the scrambled control, mouse xenografts bearing Ishikawa^TrkB^ cells overexpressing miR-204-5p showed a dramatic reduction in tumor size (*P* < 0.05) (Figure 6B and C) and tumor weight (*P* < 0.05) (Figure 6D). To verify its effects on protein expression, tumor tissue was embedded in paraffin and then stained with hematoxylin and eosin (H&E) for histological examination. As expected, TrkB and phospho-STAT3 levels were detectable in the control xenografts; however, the miR-204 expressing tumors had significantly lower levels of these proteins (Figure 6E, first four pairs of panels), thus verifying its role as a negative regulator of the TrkB/STAT3 pathway *in vivo*.

To determine whether miR-204-5p expression affects the *in vivo* proliferative ability of endometrial carcinoma cells, we examined the expression of two proliferation protein markers ki67 and PCNA by immunohistochemical staining (Figure 6E, last two pairs of panels). The ki67 proliferation index of the NC group [(92.80 ± 3.24)%] was markedly greater than that of the miR-204-5p group [(52.76 ± 9.62)%; *P* < 0.05]. Consistently, the PCNA proliferation index of the NC group [(91.30 ± 6.75)%] was significantly higher than that of the miR-204-5p group [(20.60 ± 11.46)%; *P* < 0.05], (Additional file 4: Figure S4B). These results indicate that miR-204-5p inhibits the tumorigenicity of endometrial carcinoma cells *in vivo* and further suggests a tumor-suppressive effect of miR-204-5p via the TrkB/STAT3 pathway.

### MiR-204-5p expression correlates with tumor stage and lymph node metastasis of endometrial cancer patients

**Figure 7:**
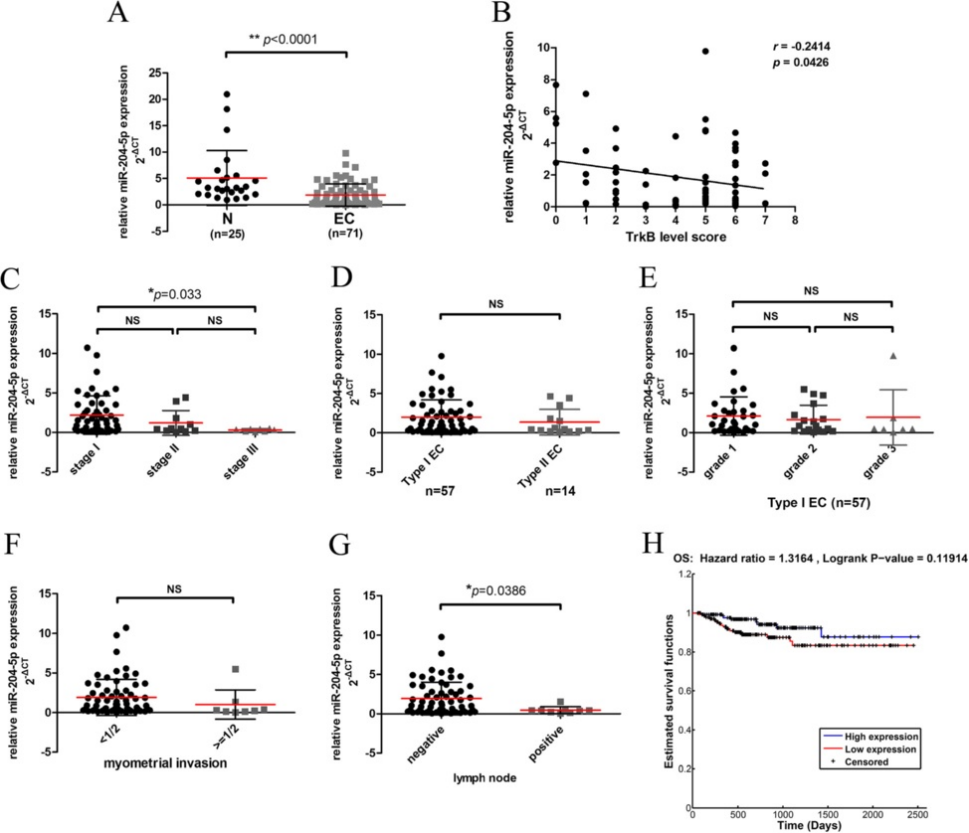
***MiR-204-5p *expression correlates with tumor stage and lymph node metastasis of endometrial cancer patients. (A)** MiR-204-5p expression in endometrial cancer tissue specimens (n = 71) and normal endometrium (N) (n = 25) was assessed by RT-PCR and normalized against U6B expression. ** *P* < 0.0001, unpaired Student’s *t* test. **(B)** Spearman correlation analysis of TrkB expression by immunohistochemistry and miR-204-5p expression by RT-PCR of 71 endometrial cancer tissue specimens. Furthermore, miR-204 expression by RT-PCR was stratified by FIGO stage **(C)**, histological type **(D)**, grade in type I **(E)**, myometrial invasion **(F)**, and lymph node status (G). *P < 0.05; NS *P* > 0.05. Bars show mean ± SD. **(H)** The Kaplan–Meier overall survival (OS) of uterine corpus endometrioid carcinoma patients (n = 279) from a dataset in the Cancer Genome Atlas stratified by miR-204-5p expression. *P* = 0.119.

To further determine the correlation between the clinicopathologic characteristics of endometrial cancer patients and miR-204-5p expression, we measured the expression levels of miR-204-5p in 25 normal endometrium samples and 71 endometrial cancer tissues by *Taq*Man PCR assays. We observed a significantly lower expression of miR-204-5p in endometrial cancer tissues compared with the normal endometrium (*P* < 0.0001) (Figure 7A). Moreover, Spearman correlation analysis showed a strong inverse correlation between the expression of TrkB and *miR-204-5p* (*r* = −0.2414, *P* < 0.05) (Figure 7B). Our RT-PCR assays further showed increasingly lower miR-204-5p levels as tumors progressed from FIGO stage I to III (stage I vs. III, *P* < 0.05) (Figure 7C). Though miR-204-5p levels were lower in type II vs. I tumors and tumors with myometrial invasion, no statistical association was observed between miR-204-5p expression and histological type, tumor grade, or myometrial invasion (*P* > 0.05) (Figure 7D-F). However, a statistically significant association was observed between miR-204-5p expression and lymph node metastasis with tumors showing positive lymph node metastasis having markedly lower levels of miR-204-5p (*P* < 0.05) (Figure 7G). Furthermore, analysis of the correlation of miR-204 expression and the overall survival (OS) of uterine corpus endometrioid carcinoma (UCEC) patients (n = 279) in an independent dataset from the Cancer Genome Atlas network further showed that though UCEC patients with high mR-204 expression exhibited a higher rate of OS, no significant difference in OS was noted between UCEC patients with high and low mR-204 expression (*P* > 0.05) (Figure 7H). However, high mR-204 expression was found to be associated with a higher likelihood of survival for UCEC patients (OS; 1.3164). These findings demonstrated that lower miR-201-5p expression is associated with advanced FIGO stages, lymph node metastasis and probably a lower chance for survival in endometrial cancer patients.

## Discussion

**Figure 8:**
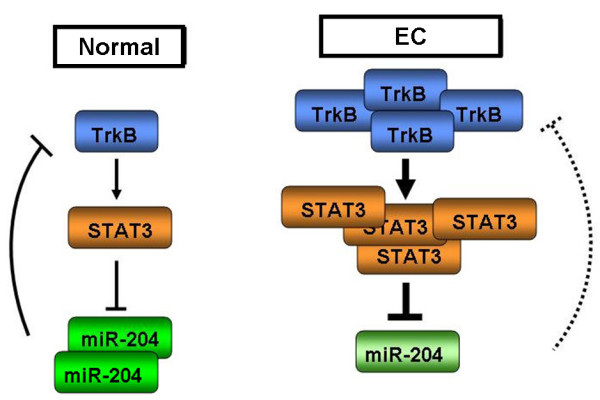
**The TrkB–STAT3–miR-204-5p regulatory circuit in endometrial cancer.** Left: In normal cells, a recurrent auto-regulatory circuit involving the expression of TrkB induces phosphorylation of STAT3 to negatively regulate the expression of miR-204-5p. MiR-204-5p, in turn, represses TrkB expression. The expression of miR-204 within this circuit maintains endometrial cells in a normal differentiated state. Right: In endometrial cancer cells, this circuit becomes dysregulated due to increased activity of the TrkB–STAT3 component of the circuit, which constitutively represses miR-204-5p. In the absence of sufficient miR-204 tumor suppressor activity, TrkB is left uncontrolled, thereby leading to carcinogenesis.

The current study identifies a novel TrkB–STAT3–miR-204-5p signaling axis that plays an important role in endometrial carcinoma growth through the accumulation of the key tumor oncogene TrkB (Figure 8). In addition, this study provides a comprehensive mechanism in the tumorigenesis of endometrial carcinoma for TrkB in inducing phosphorylation of STAT3 to regulate the expression of miR-204-5p, which, in turn, controls TrkB expression. Our results suggest that the TrkB/miR-204 pathway may serve as a novel therapeutic target for endometrial carcinoma, a disease characterized by remarkable lymph node metastasis and dismal prognosis. The information gained from this research is of important clinical implications for patients with endometrial cancer, as well as other cancer types associated with elevated TrkB expression, and may also have clinical impact on other diseases with dysregulated expression of TrkB.

Tumor dissemination and metastasis are the leading causes of death in endometrial cancer. Acquired expression of certain Trk family members (TrkA and TrkC) in various cancer types can be a good prognostic indicator [23]. However, elevated expression of TrkB has been associated with poor survival of breast cancer patients [24]. A role for TrkB in preventing anoikis was revealed in an unbiased screen of rat intestinal epithelial cells [10] and TrkB was shown to induce a profound morphological change in which the expression of epithelial markers was reduced and the expression of mesenchymal markers was enhanced [25,26]. TrkB is proposed to function as a key regulator of oncogenesis and tumor progression in a variety of human cancers, including lung [7], breast [8], pancreatic [27], stomach [28], colon [28,29], prostate [30], and ovarian cancer [31]. In our previous study, we confirmed that stimulation with brain-derived neurotrophic factor (BDNF), a natural ligand for TrkB, enhances TrkB-mediated endometrial carcinoma cell survival. The kinase activity of TrkB contributes to endometrial carcinoma progression by inhibiting anoikis and promoting EMT [9]. In the present study, *TrkB* mRNA appeared to have no obvious change between endometrial carcinoma and normal tissues while TrkB protein levels varied markedly between endometrial carcinoma and normal tissues. This observation led us to hypothesize that TrkB expression is upregulated via a posttranscriptional mechanism in endometrial carcinoma progression.

Epigenetic effects are a key aspect of the relationship between miRNAs and carcinogenesis [32]. Dysregulated expression of miRNAs has recently been associated with carcinogenesis in endometrial carcinoma [18,33,34]. Oncogenesis may involve complex patterns of regulation because tumor suppressor miRNAs themselves are subject to transcription factor regulation, in addition to their epigenetic regulation of oncogenes or tumor suppressor genes [35]. These findings provide a new perspective on TrkB-induced metastasis. In the present study, we explored TrkB-dependent regulation of miRNA expression in two pairs of endometrial carcinoma cell lines via comprehensive miRNA profiling analysis. We provide evidence that miR-204-5p, which acts as a potent tumor growth and metastasis suppressor both *in vitro* and *in vivo*, is somatically lost in human endometrial carcinoma. The finding that miR-204-5p is downregulated in endometrial carcinoma is intriguing, as decreased miR-204-5p levels have been reported in several types of solid tumors [36-38], and suggests that loss of miR-204-5p may be a common event in tumorigenesis.

STAT3 is classified as a proto-oncogenic transcription factor, and constitutive activation of STAT3 has been frequently detected in various types of human cancers including endometrial carcinoma [39]. We and others have shown that as a receptor tyrosine kinase, TrkB activates downstream signaling cascades that ultimately induce cellular proliferation through the STAT3 signaling pathways [20]. To date, many studies have focused on miRNAs and their regulated targets, but few studies have investigated how miRNA expression is controlled transcriptionally [40]. In this study, we show that reduction of miR-204 correlates with phosphorylation of STAT3, which directly binds to the regulatory sites of its host gene, *TRPM3*, suggesting a regulatory mechanism for TrkB in controlling miR-204 levels through STAT3 activation. Interestingly, TRPM3 is a member of the transient receptor potential melastatin (TRPM) family, which has also been reported to be associated with cancer progression. Downregulation of *TRPM1/melastatin* mRNA in primary malignant melanoma is a prognostic marker for metastasis [41]; however, the pathobiologic role of TRPM3 in endometrial carcinoma remains largely unknown. Whether TRPM3 and miR-204 could cooperate with each other in the pathogenesis of human endometrial carcinoma remains unknown but is an intriguing and biologically important question. Nevertheless, these data suggest that TrkB-dependent STAT3 activation is an important event in regulating miR-204 transcription in endometrial cancer cells and possibly other cancer types.

MiR-204 has been previously shown to be greatly downregulated in endometrioid adenocarcinoma tissues by human miRNA microarray [18]. In contrast, a more recent study showed that miR-204 is upregulated in the serum of endometrial carcinoma patients [42]. Therefore, the regulation of miR-204 is complex. Our results are consistent with the former study in showing that miR-204-5p expression in endometrial carcinoma tissues is significantly lower than that in the normal endometrium. Furthermore, we present the first direct evidence that reduced expression of miR-204-5p is significantly associated with lymph node metastasis. Our results support the possibility that miR-204-5p may constitute a potential biomarker for good prognosis of endometrial cancer, and therapeutic approaches targeting elevated levels of miR-204-5p should be explored as a novel approach to improve the clinical outcomes of endometrial carcinoma patients.

The characterization of miR-204-5p function, to date, has been limited, although several mRNA targets have been identified that are important in normal cell development, including MEIS1, HOXA9, MEIS2, RUNX2, SIRT1 and Mcl-1 [43-47]. MiR-204-5p has been reported to act as a tumor suppressor in a variety of cancers through different mechanisms. MiR-204 also targets forkhead box C1 (FOXC1), which regulates metastasis and invasion in human endometrial cancer-derived HEC-1A cells. In endometrial cancer, decreased expression of miR-204 causes dysfunctional regulation of FOXC1, which results in enhanced metastasis and invasion of tumor cells [19]. Recently, miR-204 has been suggested as a novel predictor of outcome in neuroblastoma, functioning, at least in part, by increasing the sensitivity to cisplatin through direct targeting and downregulation of anti-apoptotic BCL2 and TrkB [48]. Consistent with the study, we determined that miR-204-5p specifically targets the 3′UTR of TrkB, resulting in a significant reduction of full-length TrkB protein. Our results, therefore, provide evidence for another distinct role of miR-204 in carcinogenesis and further highlight the importance of miR-204 expression in endometrial carcinoma. Interestingly, TrkB has also been identified as a target of miR-200c, which was markedly upregulated in endometrial carcinoma [49]. A TrkB construct that is resistant to miR-200c is unable to induce anoikis [50], which may explain our observation that there was no apparent change in *TrkB* mRNA transcript levels between endometrial carcinoma and normal tissues. Further investigation is required to elucidate the underlying mechanisms of miR-200, given that downstream components of TrkB signaling, such as Twist and Snail 1, are also targets of the miR-200 family [51]. However, it will be of interest to also determine the potential functional contribution of miR-200 within the TrkB-STAT3-miR-204-5p axis.

## Conclusions

Overall, this study uncovers a novel regulatory circuitry involving TrkB–STAT3–miR-204-5p that is critical to the tumorigenicity of human endometrial carcinoma and indicates that reestablishing miR-204-5p expression could be explored as a potential new therapy for this disease.

## Materials and methods

### Acquisition of tissue specimens

Primary tumor tissue samples were acquired from 110 treatment-naïve endometrial carcinoma patients who underwent hysterectomy with lymph node dissection at our institution between August 2009 and April 2012. The resected specimens were histologically examined by H&E and immunohistochemical staining. Among them, primary fresh tissues were collected from 71 of 110 corresponding patients immediately after surgical removal and snap-frozen in liquid nitrogen until further use. Patient demographic and baseline characteristics are shown in Table 2. In addition, 25 normal endometrium samples were obtained from patients who underwent hysterectomy due to other diseases than endometrial carcinoma. Tumor stage and grade were established according to the 2009 Federation International of Gynecology and Obstetrics (FIGO) surgical staging system [52]. 110 formalin-fixed, paraffin-embedded tissues were used for immunohistochemistry, and 71 fresh frozen samples from the same patients were used for LCM/qRT-PCR analysis. Acquisition of tissue specimens was approved by the Human Investigation Ethical Committee of the authors’ affiliated institution.

**Table 2:** Demographic and baseline characteristics of endometrial carcinoma patients

**Variable**	**N(%)**
**Total**	71(100)
**Age (years)**	
50	14(19.8)
50	57(80.2)
**FIGO stage**	
I	60(84.5)
II	7(9.8)
III	4(5.7)
IV	0(0)
**Grade (Endometrioid, n** = **57)**	
G1	32(56.1)
G2	18(31.6)
G3	7(12.3)
**Histological type**	
Endometrioid	57(80.3)
Non-endometrioid	14(19.7)
**Myometrial invasion**	
<1/2	67(94.4)
≥1/2	4(5.6)
**Lymph node metastasis**	
Negative	63(88.7)
Positive	8(11.3)

### Laser capture microdissection (LCM)

Ten to 20 serial frozen sections of 8 μm thickness were fixed in 70% ethanol for 2 min at −20°C and stained with HistoGene using a frozen section staining kit (Applied Biosystems, Foster City, CA). Then, the sections were rinsed in ice-cold RNA nuclease-free water at −20°C followed by incubation in xylene for 2 min at −20°C. After the sections were air-dried, the targeted cells were microdissected according to a UV cutting and laser capture procedure using the LCM system (Lecia, LMD 7000, Germany). Endometrial cancer cells and normal epithelial cells were captured onto CapSureMacro LCMcap (Applied Biosystems, Foster City, CA) to allow analysis of differential expression between cancer cells and normal endometrial cells.

### Cells and transfections

Human endometrial cancer cell lines Ishikawa and HEC-1B and human embryonic kidney 293 T cells were obtained from the Chinese Academy of Sciences Committee Type Culture Collection (Shanghai, China). Ishikawa^TrkB^ cells stably expressing ectopic *TrkB* and HEC-1B^shTrkB^ cells stably expressing *TrkB*-specific short hairpin RNA (shRNA) were previously described [9]. Cells were maintained at 37°C in a humidified atmosphere containing 5% CO_2_ in Dulbecco’s modified Eagle’s medium/F12 (Gibco, Auckland, NZ) supplemented with 10% fetal bovine serum (FBS) (Invitrogen, Carlsbad, CA).

For transfections, cells were seeded at 2 × 10^5^ cells/well in 6-well plates and after growth overnight were transfected with miR-204i, miR-204 m, miR-204 mimic negative control (miR-204 m NC), or miR-204 inhibitor negative control (miR-204i NC) (all from GenePharma, Shanghai, China) using Lipofectamine^2000^ (Invitrogen, Carlsbad, CA, USA) according to the manufacturer’s instructions. For siRNA knockdown of *TrkB or STAT3*, we used siRNA against *TrkB* (siTrkB) [53], or siRNA against *STAT3* (siSTAT3) [54]. A scrambled siRNA (siTrkB-NC) was used as control. The sequences are listed in Table 3. Ishikawa cells were co-transfected with miR-204i and siTrkB, or miR-204i and siTrkB-NC using Lipofectamine^2000^.

**Table 3:** Oligonucleotide sequences used in the study

**Identifier**	**Sense primer sequences**	**Antisense primer sequences**
**miR-204i**	5′-AGGCAUAGGAUGACAAAGGGAA-3′	
**miR-204i NC**	5′-CAGUACUUUUGUGUAGUACAA-3′	
**miR-204 m**	5′-UUCCCUUUGUCAUCCUAUGCCU-3′	5′-GCAUAGGAUGACAAAGGGAAUU-3′
**miR-204 m NC**	5′-UUCUCCGAACGUGUCACGUTT-3′	5′-ACGUGACACGUUCGGAGAATT-3′
**siTrkB**	5′-GAAUUGACGAUGGUGCAAATT-3′	5′-UUUGCACCAUCGUCAAUUCCA-3′
**siTrkB-NC**	5′-UUCUCCGAACGUGUCACGUTT-3′	5′-ACGUGACACGUUCGGAGAATT-3′
**siSTAT3**	5′-GGGACCUGGUGUGAAUUAUTT-3′	5′-AUAAUUCACACCAGGUCCCTT-3′
**TrkB-3′UTR-WT**	5′-GCTCTAGATTTTGGCATTATCTCTTTCTCT-3′	5′-GCTCTAGATTGTTCCTCCCATATTGCG-3′
**TrkB-3′UTR-MT**	5′-GACAACAAATATTTCACTTAA-3′	5′-CTTTGATGTGGATGAAAAACGGTTACAAC-3′
**GV219 STAT3 vector**	5′-CGCAAATGGGCGGTAGGCGTG-3′	5′-CCCACTGTCCTTTCCTAATAA-3′
**GV254-hsa-miR-204**	5′-AGCTGTACAAGTAAGCCTGATCATGTACCCATAGG-3′	5′-GGGAGAGGGGCTTAGCTTATGGGACAGTTATGGGC-3′

### U0126 treatment

Cells were seeded at 1.0 × 10^5^ cells per well of a 12-well plate, containing 1.2 mL regular medium. The media was then changed to phenol-red free medium with 0.5% stripped FBS for incubation at 37°C overnight. Immediately prior to treatment, the medium in the culture plates was aspirated, triply washed with PBS and replaced with fresh medium. U0126 (Cell Signaling Technology, Danvers, MA) dissolved in DMSO was subsequently added to each well. Concurrently, the same amount of DMSO was added to the control wells.

### MiRNA microarray

Total cellular RNA was isolated using a mirVana miRNA extraction Kit (Ambion, Austin, TX) according to the manufacturer’s instruction and labeled and hybridized using the Human MicroRNA Microarray Kit (Agilent Technologies, Santa Clara, CA) according to the manufacturer’s protocol. Hybridization signals were detected with an Agilent DNA microarray scanner G2505C, and scan images were analyzed using Agilent feature extraction software (v10.7.3). Data were analyzed using GeneSpring GX 12.0 software (Agilent Technologies). Values < 0.01 were set to 0.01 and each measurement was divided by the 75^th^ percentile of all measurements from the same samples. MiRNAs whose expression differed by at least 1.5-fold between Ishikawa and Ishikawa^TrkB^ cells (or between HEC-1B and HEC-1B^shTrkB^) were selected.

### Quantitative real-time RT-PCR

Total cellular RNA was extracted using Tri-reagent (Molecular Research Center; Cincinnati, OH). For miRNA analysis, mature miRNA was reverse-transcribed from total RNA using a *Taq*Man MicroRNA Reverse Transcription kit. Real-time PCR was performed according to the manufacturer’s instructions. MiRNA expression was determined using the 2(^-△Ct^) method and normalized against U6B. For quantification of *TrkB* and *TRPM3*, cDNA was generated using a Prime Script RT reagent Kit (TaKaRa; Dalian, China), and real-time PCR was performed on an ABI Prism 7000 Sequence Detection System with SYBR Premix Ex Taq (TaKaRa). The primer sequences are listed in Table 4. All experiments were performed in triplicate at least three times independently.

**Table 4:** Primers used for quantitative real-time PCR analysis

**mRNA**	**Primer sequence**
**NTRK2(TrkB)**	Forward 5′-GGGACACCACGAACAGAAGTA-3′
	Reverse 5′-ACCACAGCATAGACCGAGAGA-3′
**TRPM3**	Forward 5′-GGAAAGGGCTCATCAAAGCAG-3′
	Reverse 5′-CCAACATGACGAATAACACCTGT-3′
**TRPM3 UP1**	Forward 5′-AACTCATCCCTGGAAGCAAACTGC-3′
	Reverse 5′-TTTGGGCCTCAAGGAAGCAAACTG-3′
**TRPM3 DW1**	Forward 5′-ATGTTCCAGGAAGAGGGAACAGCA-3′
	Reverse 5′-TTCTACCCAGAACCTTCCTTCCCA-3′
**TRPM3 DW2**	Forward 5′-AAGGAAGTGACTCACAGGAAGGCA-3′
	Reverse 5′-GGCTTGCTGTTGCCCTTGGATAAA-3′
**VEGF**	Forward 5′-CATACGTGGGCTCCAACAGG-3′
	Reverse 5′-CGGAGAAGCTGTGTGGTTCCG-3′
**β-actin**	Forward 5′-CAGCCATGTACGTTGCTATCCAGG-3′
	Reverse 5′-AGGTCCAGACGCAGGATGGCATG-3′

### Chromatin immunoprecipitation (ChIP)-PCR

Chromatin immunoprecipitation (ChIP) assays were performed as previously described [22] using anti-phospho-STAT3 antibody (Tyr705) or rabbit isotype IgG (both from Cell Signaling Technology, Danvers, MA). STAT3-binding sites surrounding *TRPM3* were predicted using *in silico* analysis (University of California, Santa Cruz Genome Browser and ENCODE database) as depicted previously [22] and by quantitative real-time PCR using SYBR green (Takara). Enrichment was calculated using the comparative Ct method and primers used are shown in Table 4 and were analyzed for specificity, linearity range, and efficiency to accurately evaluate occupancy (percentage of immunoprecipitation/input). *VEGF* was used as positive control and *IgG* as negative control.

### Cell proliferation and clonogenic assays

For cell proliferation studies, cells were plated at 1 × 10^3^ cells/well in 96-well plates after 24-h serum starvation. Cell viability was determined at the appropriate time points using the 3-(4,5-dimethylthiazol-2-yl)-2,5-diphenyltetrazolium (MTT) assays with commercially available kits (Sigma; St. Louis, MO). Absorbance was measured at 490 nm using a Spectra Max 190 microplate reader (BIO-RAD; Hercules, CA). For clonogenic survival assays, cells were seeded at 2 × 10^3^ cells/plate in 6 well plates. After 2 weeks, cell colonies were stained with 0.1% crystal violet and counted.

### Cell migration and invasion assays

Cells were plated serum-free at a density of 2 × 10^5^/well in invasion chambers (8 μm pore size; BD Biosciences, San Jose, CA) with or without Matrigel-coating. Medium containing 10% FBS was added into 24-well plates as a chemoattractant. After 6 h (migration assay) or 24 h incubation (invasion assay), cells were fixed with 4% paraformaldehyde for 1 h. Cells on the apical side of each insert were removed by mechanical scraping. Cells that migrated to the basal side of the membrane were stained with 0.1% crystal violet, visualized and counted under a Leica DMI 3000B microscope at 200 × magnification.

### Immunoblotting assays

For Western blotting assays, conducted as previously depicted [9], antibodies against the following proteins were used: TrkB and phospho-TrkB (both from Abcam), phospho-P44/42 MAPK (thr204/tyr204), P44/42 MAPK, phospho-JAK2 (tyr1007/1008), JAK2, phospho-STAT3 (tyr705) and STAT3 (all from Epitomics). Protein bands were visualized by enhanced chemiluminescence (Pierce Biotechnology) and protein expression was normalized against β-actin.

### Luciferase assays

A DNA fragment containing a partial wildtype or mutant 3′UTR of *TrkB* was cloned into the pMIRGLO-REPORT luciferase vector (Ambion) and the resultant vectors were designated pMIRGLO-TrkB-3′UTR-WT and pMIRGLO-TrkB-3′UTR-MT, respectively. We performed the luciferase assays using 293 T cells transiently transfected with Renilla constructs (as an internal control) or plasmids pMIRGLO-TrkB-3′UTR-WT or pMIRGLO-TrkB-3’UTR-MT with or without miR-204 m or miR-204 m NC using the Dual Luciferase Assay system following the manufacturer’s instructions (Promega, Madison, WI). All luciferase activity readings were normalized relative to the activity of the Renilla luciferase control and the results were expressed as relative luciferase activity (Firefly LUC/Renilla LUC). All experiments were performed in triplicate at least 3 times independently.

### Generation of stable miR-204-5p expressing Ishikawa^TrkB^ cell lines

The lentivirus vector expressing *miR-204-5p* was prepared using the Lenti-miR-204 miRNA Precursor Expression Construct according to the manufacturer’s protocol (Genechem, Shanghai) (Additional file 4: Figure S4A). Stable Ishikawa^TrkB^ cells containing the lentivirus vector carrying *miR-204* or scramble hairpin control (Genechem) were established after selection with appropriate antibiotics.

### Xenograft assays

For xenograft experiments, sixteen 5-week old female BALB/c nude mice (Chinese Academy of Sciences, Shanghai, China) were injected subcutaneously with 5 × 10^6^ Ishikawa^TrkB^ miRNA NC and Ishikawa^TrkB^ miR-204 cells, respectively, in the nape. Tumor size was monitored every 4 days by measuring the length and width with calipers, and tumor volumes were calculated with the formula: (L × W^2^) × 0.5 mm^3^, where L is the length and W is the width of each tumor. At the completion of the experiment, mice were sacrificed and the tumors were weighed, dissected, measured and photographed. The study protocol was approved by the local institution review board. The mice used in this experiment were maintained under specific pathogen-free conditions and handled in accordance with the NIH Animal Care and Use Committee regulations.

### Immunohistochemistry and *in situ* hybridization

Paraffin embedded endometrial cancer and normal endometrium tissue sections (4 μm) were processed for immunohistochemistry as previously described [9]. Briefly, after deparaffinization and dehydration, specimens were boiled in 10 mM sodium citrate buffer to unmask antigens. Specimens were then blocked and incubated with primary antibody overnight at 4°C. Antibody binding was detected using Envision reagents (Boster bioengineering, Wuhan, China) according to the manufacturer’s instructions. For evaluation of TrkB expression, staining intensity was scored as 0 (negative), 1 (weak), 2 (medium), or 3(strong). The extent of staining was scored as 0 (0%), 1 (1%–25%), 2 (26%–50%), 3 (51%–75%), or 4 (76%–100%), according to the percentage of the positively stained areas in relation to the whole tumor area. The sum of the intensity score and the extent score was used as the final staining score (0–7) [55]. The results were assessed by two pathologists who were blinded to details regarding patient background.

Additionally, tissue sections (4 μm) of mouse xenografts were routinely prepared and immunohistochemistry was performed using standard avidin-biotin techniques. Anti-TrkB antibody (Abcam) and anti-phospho-STAT3 antibody (Epitomics) were used for the procedure. Brown staining of nuclei was regarded as positive. For in situ hybridization, sections of mouse xenografts were dewaxed and rehydrated, followed by digestion with proteinase K. A 5′ digoxin-labeled locked nucleic acid-modified miR-204-5p probe (Ambion Life Technologies,) was incubated on coverslips at 37°C overnight. Then, the sections were incubated with anti-digoxin antibody (Boster) at 37°C for 1 hour, followed by staining with nitro blue tetrazolium°-bromo-4-chloro-3-indolylphosphate. MiRNA-204-5p in the cytoplasm was stained purple.

### *In silico* analysis of miR-204 and OS of UCEC patients

We performed an in silico analysis of the association between miR-204 and OS of UCEC patients using published data from the Cancer Genome Atlas network [56] (Additional file 5). Clinical information was downloaded from the Additional file 5 “datafile.S1.1.KeyClinicalData.xls” and miRNA expression profiling by miR-seq was downloaded from the Additional file 5 “bcgsc.ca_UCEC.Illumina_miRNASeq.tar” [56]. The RPKM value of miRNAs in each sample was used to construct the expression profile for hsa-miR-204 and the median of miR-204 expression was used as the cutoff value for high and low expression of miR-204.

### Statistical analysis

Data were presented as mean ± SD and analyzed by the SPSS 16.0 software (SPSS Inc., Chicago, IL). Student’s *t*-test was used for comparison between two groups, and one-way ANOVA followed by post-hoc Turkey’s test was used for comparison among multiple groups. Correlation was analyzed by Spearman test, and OS was assessed using standard log-rank test and by the Kaplan-Meier method. All *P*-values are two-sided, and *P*-values less than 0.05 indicated statistically significant difference. Each experiment was performed as least three times independently.

## Abbreviations

ChIP: Chromatin immunoprecipitation; EMT: Epithelial to mesenchymal transition; FIGO: International Federation of Obstetrics and Gynecology; FOXC1: Forkhead box C1; H&E: Hematoxylin and eosin; IHC: Immunohistochemistry; LCM: Laser capture microdissection; NTRK: Neurotrophic tyrosine receptor kinase; PCR: Polymerase chain reaction; RT: Reverse transcription; siRNA: Small interfering RNA; TCGA: The Cancer Genome Atlas; TRPM: Transient receptor potential melastatin; UCEC: Uterine corpus endometrioid carcinoma.

## Competing interests

The authors declare that they have no competing interests.

## Authors’ contributions

WB, HHW, LHW and XPW conceived and designed the experiments. WB, HHW, FJT, XYH and MTQ performed the experiments: WB, FJT and JYW analyzed the data. WB and HHW wrote the manuscript. All authors read and approved the final manuscript.

## Supplementary Material

Additional file 1: Figure S1 Predicted miR-204 binding site in the *TrkB* 3′UTR. **(A)** Alignment of the predicted miR-204 binding site in the *TrkB* 3′UTR across five different species. The seed sequence is shaded in white (upper panel) or labeled in blue (lower panel). **(B)** 293 T cells were co-transfected with pMIRGLO-TrkB-3′UTR-MT and STAT3 vector (GV219) or empty vector. Luciferase activities are shown as mean ± SD of at least three independent experiments done in triplicate. NS, *P* > 0.05. Click here for file

Additional file 2: Figure S2 Effect of MAPK pathway inhibition on miR-204-5p expression. **(A)** Ishikawa^TrkB^ and HEC-1B cells were treated with MAPK inhibitor U0126 (10 μM) or DMSO (control), and phospho-P44/42 were assessed by Western blotting assays. **(B** and **C)** Inhibition of P44/42 MAPK has no effect on *miR-204-5p* expression in Ishikawa^TrkB^ and HEC-1B cells. Data are expressed as mean ± SD of at least three independent experiments. NS, *P* > 0.05. Click here for file

Additional file 3: Figure S3 TrkB promotes while miR-204-5p suppresses the clonogenic growth, migration and invasion of endometrial cancer cells. **(A)** Upper and lower panel shows representative images of colony formation in Ishikawa^TrkB^ and HEC-1B^shTrkB^ cells. **(B)** Migrated cells on the lower surface of the Transwell filter were stained and photographed, 200×. **(C)** Invasive cells on the lower surface of the Transwell filter were stained and photographed, 200 × . Click here for file

Additional file 4: Figure S4 Structure of the miR-204 plasmid and the proliferation index in xenograft tumor tissues. **(A)** Structure of the miR-204 lentiviral plasmid and its inserted primer sequence. **(B)** Ki67 and PCNA protein expression in the nuclei of tumor cells transduced with this plasmid. Bars show mean ± SD. **P < 0.01. Click here for file

Additional file 5 Clinical patient information conducted by The Cancer Genome Atlas (TCGA) network. Click here for file
