# Supplementary figures and images for: A TrkB–STAT3–miR-204-5p regulatory circuitry controls proliferation and invasion of endometrial carcinoma cells
Source: Mol Cancer. 2013 Dec 9;12:155. doi: 10.1186/1476-4598-12-155 (PMC3879200; doi:10.1186/1476-4598-12-155)

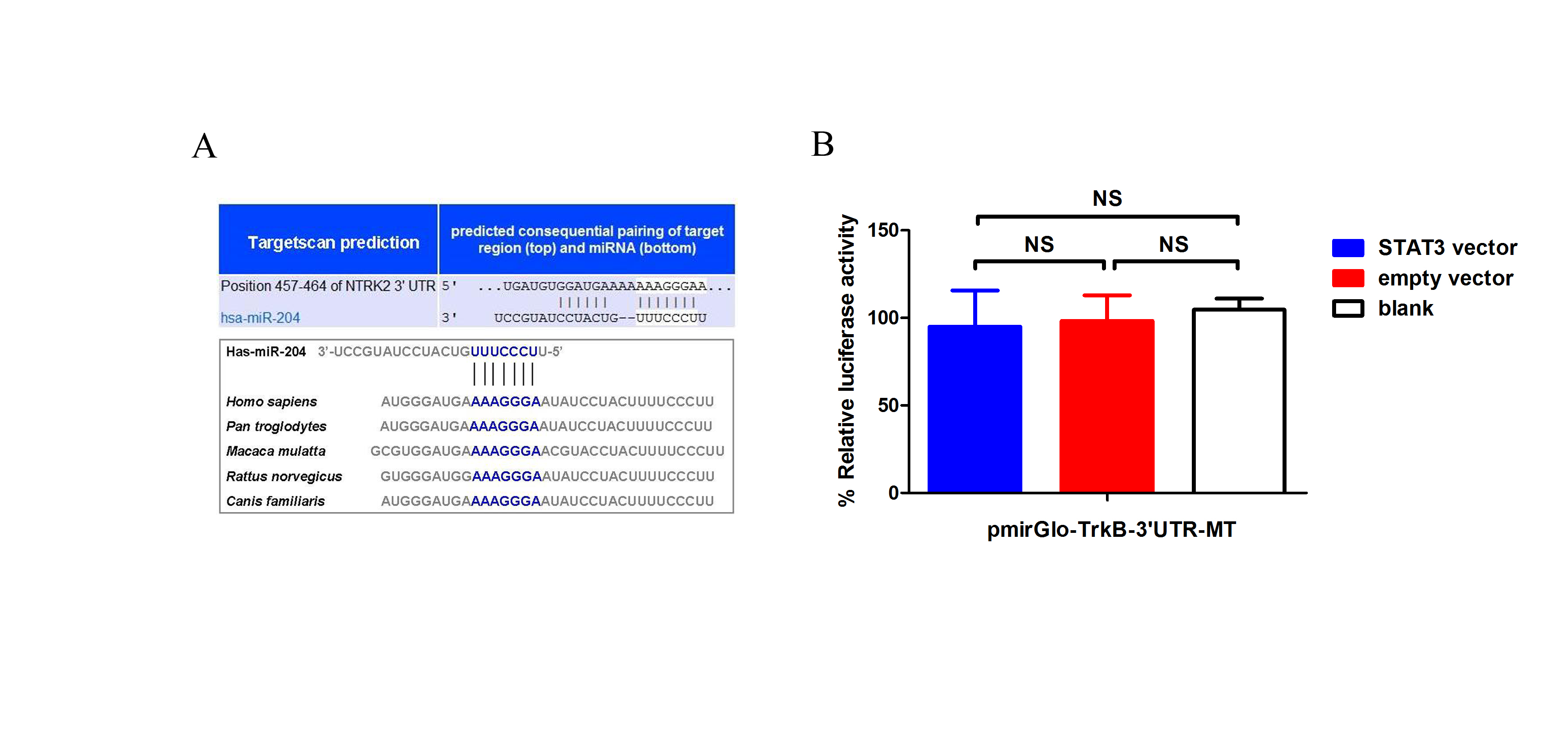

Supplement: Additional file 1: Figure S1 — Predicted miR-204 binding site in the TrkB 3′UTR. (A) Alignment of the predicted miR-204 binding site in the TrkB 3′UTR across five different species. The seed sequence is shaded in white (upper panel) or labeled in blue (lower panel). (B) 293 T cells were co-transfected with pMIRGLO-TrkB-3′UTR-MT and STAT3 vector (GV219) or empty vector. Luciferase activities are shown as mean ± SD of at least three independent experiments done in triplicate. NS, P > 0.05. [file 1476-4598-12-155-S1.tiff]

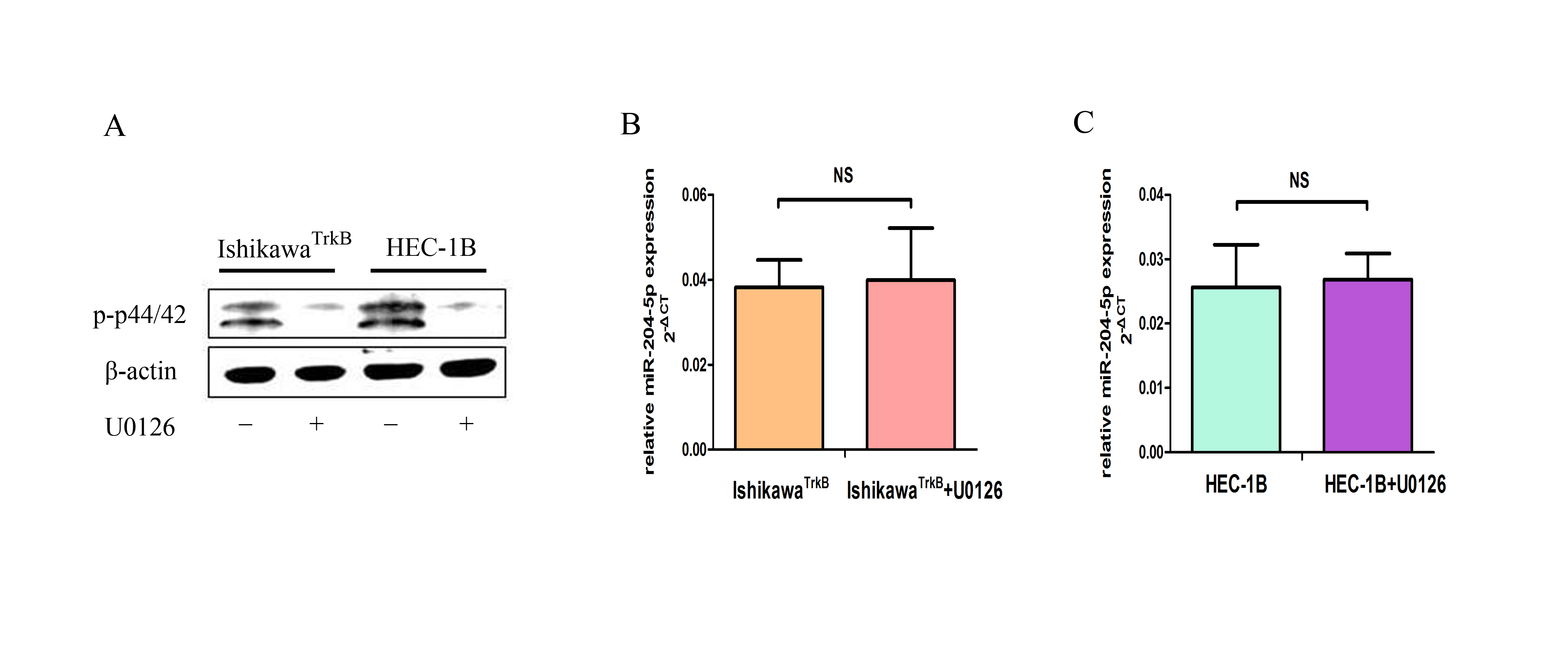

Supplement: Additional file 2: Figure S2 — Effect of MAPK pathway inhibition on miR-204-5p expression. (A) IshikawaTrkB and HEC-1B cells were treated with MAPK inhibitor U0126 (10 μM) or DMSO (control), and phospho-P44/42 were assessed by Western blotting assays. (B and C) Inhibition of P44/42 MAPK has no effect on miR-204-5p expression in IshikawaTrkB and HEC-1B cells. Data are expressed as mean ± SD of at least three independent experiments. NS, P > 0.05. [file 1476-4598-12-155-S2.tiff]

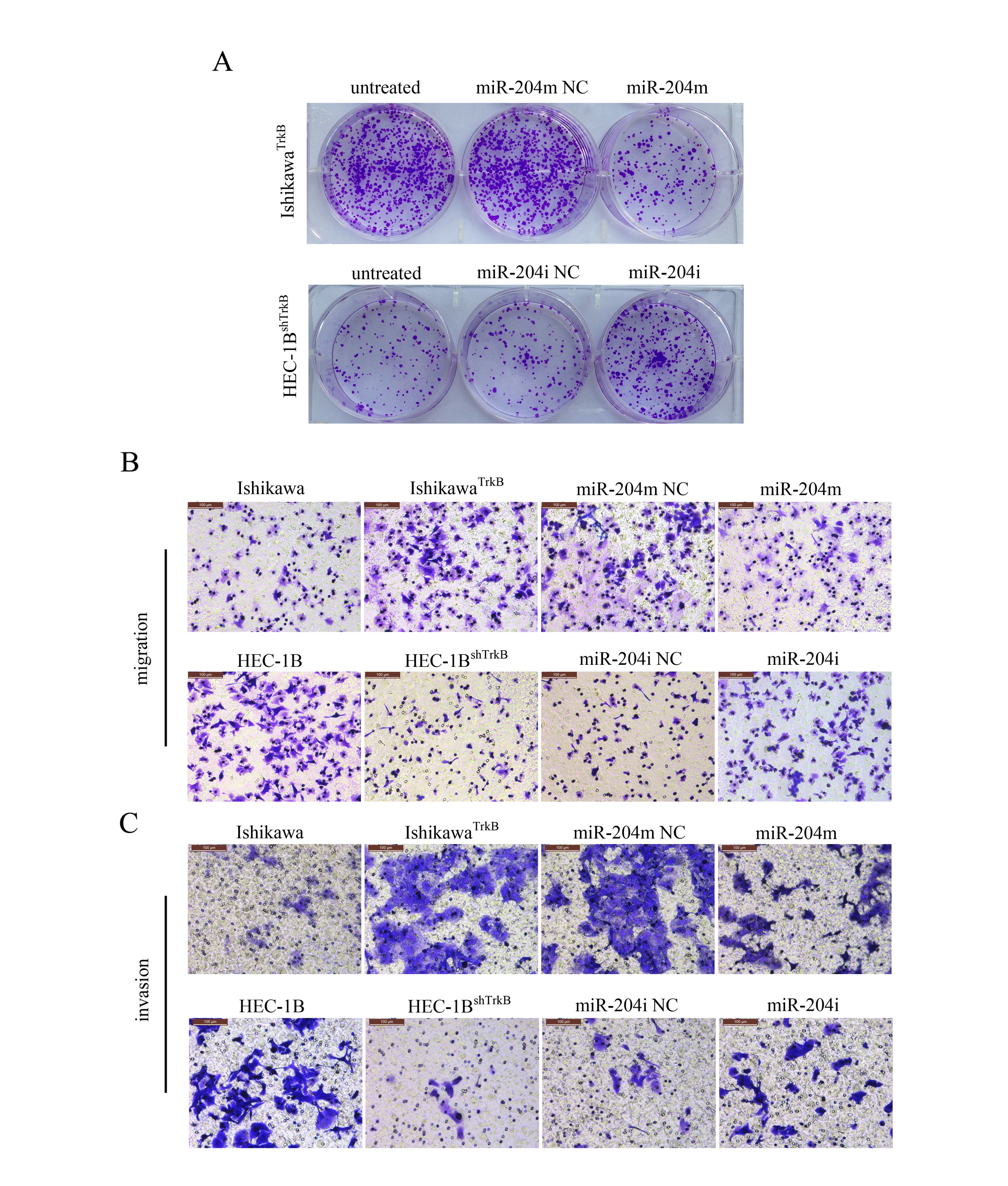

Supplement: Additional file 3: Figure S3 — TrkB promotes while miR-204-5p suppresses the clonogenic growth, migration and invasion of endometrial cancer cells. (A) Upper and lower panel shows representative images of colony formation in IshikawaTrkB and HEC-1BshTrkB cells. (B) Migrated cells on the lower surface of the Transwell filter were stained and photographed, 200×. (C) Invasive cells on the lower surface of the Transwell filter were stained and photographed, 200 × . [file 1476-4598-12-155-S3.tiff]

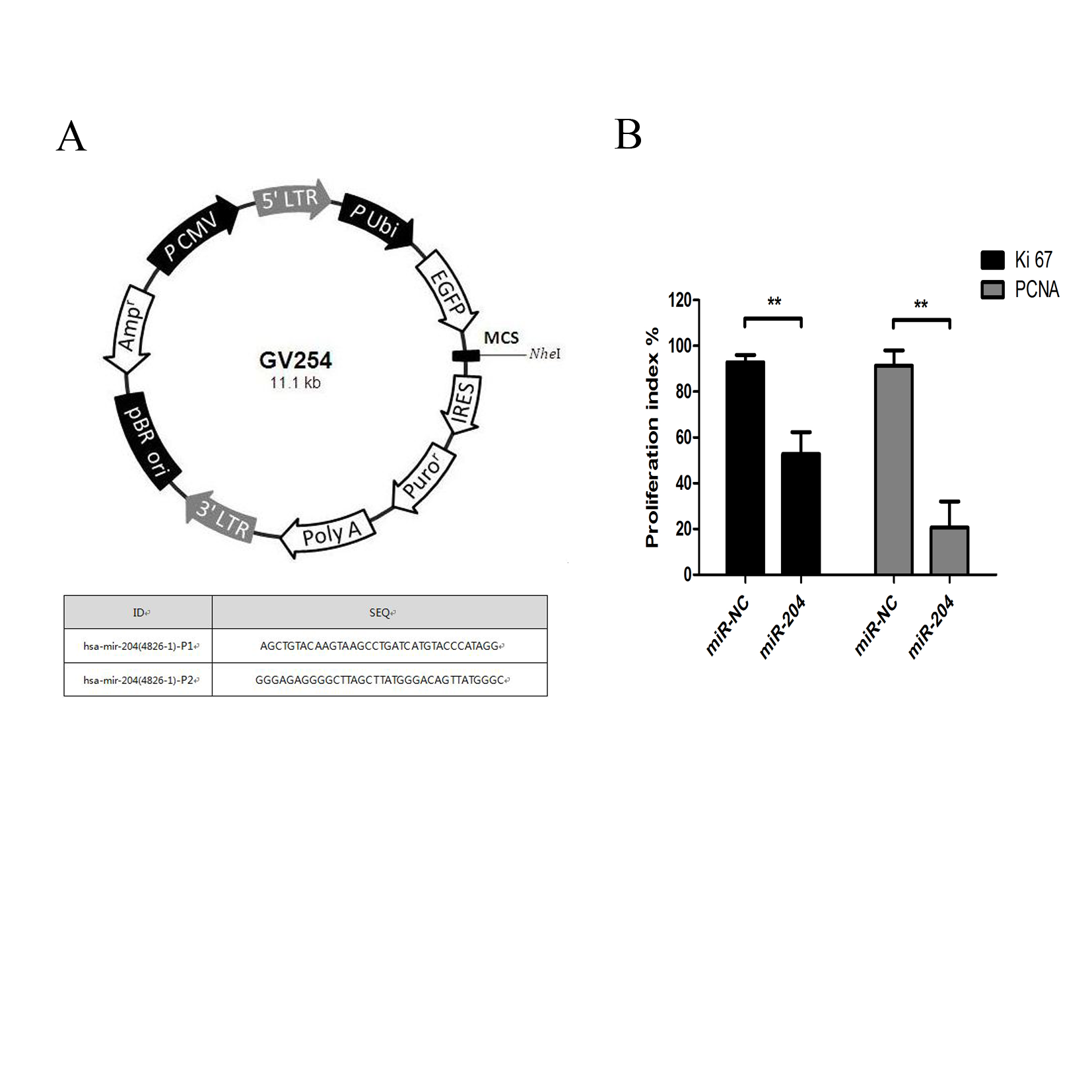

Supplement: Additional file 4: Figure S4 — Structure of the miR-204 plasmid and the proliferation index in xenograft tumor tissues. (A) Structure of the miR-204 lentiviral plasmid and its inserted primer sequence. (B) Ki67 and PCNA protein expression in the nuclei of tumor cells transduced with this plasmid. Bars show mean ± SD. **P < 0.01. [file 1476-4598-12-155-S4.tiff]
